# Age‐related variations in diffuse axonal injury following mild traumatic brain injury

**DOI:** 10.1002/alz.093862

**Published:** 2025-01-09

**Authors:** Marangelie Criado‐Marrero, Sakthivel Ravi, Ekta Bhaskar, Marcelo Febo, Jose F. F Abisambra

**Affiliations:** ^1^ University of Florida, Gainesville, FL USA

## Abstract

**Background:**

Repetitive mild traumatic brain injury (rmTBI) represents a substantial health challenge, urging a more thorough investigation into its early effects and possible interventions. The collective consequences of rmTBI encompass various neurobiological and neuropsychological impairments, increasing susceptibility to diseases like Alzheimer’s and related dementias. Employing the Closed‐Head Impact Model of Engineered Rotational Acceleration (CHIMERA) approach for TBI induction, our prior study revealed connectivity alterations within 53% of regions in young and aged wild‐type mice five days post‐injury.

**Method:**

Given rmTBI’s potential for causing diffuse axonal injury, the current study utilized advanced neuroimaging techniques—Diffusion Tensor Imaging (DTI) and Neurite Orientation Dispersion and Density Imaging (NODDI)—to detect white matter damage in 36 regions of the brain.

**Result:**

Major age‐dependent disruptions were observed in the corticospinal tract, hippocampal commissure, anterior cingulate, and optic tract. Surprisingly, mean diffusivity (MD), a reflection of overall water diffusion, showed unique age‐related effects. Three main regions were affected by injury in young mice: the entorhinal cortex, anterior cingulate, and optic tract. Regardless of injuries, aged animals exhibited an inverse correlation between fractional anisotropy (FA) and radial diffusivity (RD) in the secondary motor area, corticospinal tract, and hippocampal commissure.

**Conclusion:**

Our discoveries are significant for revealing age‐specific changes and understanding the intricate connection between age and injury, shaping brain tissue integrity, particularly in the context of aging research.

